# Comparative mRNA and microRNA Expression Profiling of Three Genitourinary Cancers Reveals Common Hallmarks and Cancer-Specific Molecular Events

**DOI:** 10.1371/journal.pone.0022570

**Published:** 2011-07-25

**Authors:** Xianxin Li, Jiahao Chen, Xueda Hu, Yi Huang, Zhizhong Li, Liang Zhou, Zhijian Tian, Hongyu Ma, Zhiyun Wu, Maoshan Chen, Zujing Han, Zhiyu Peng, Xiaokun Zhao, Chaozhao Liang, Yong Wang, Liang Sun, Jing Chen, Jun Zhao, Binghua Jiang, Huanming Yang, Yaoting Gui, Zhiming Cai, Xiuqing Zhang

**Affiliations:** 1 Department of Urology, Peking University Shenzhen Hospital, Shenzhen, China; 2 Guangdong Key Laboratory of Male Reproductive Medicine and Genetics, Peking University Shenzhen Hospital, Shenzhen, China; 3 Beijing Genomics Institute at Shenzhen, Shenzhen, Guangdong, China; 4 School of Bioscience and Biotechnology, South China University of Technology, Guangzhou, Guangdong, China; 5 Beijing Institute of Genomics, Chinese Academy of Sciences, Beijing, China; 6 Graduate University of Chinese Academy of Sciences, Beijing, China; 7 Department of Urology, The Second Xiangya Hospital of Central South University, Changsha, China; 8 Department of Urology, The First Affiliated Hospital of Anhui Medical University, Hefei, China; 9 Shantou University Medical College, Shantou, China; 10 Department of Urology, The Second People's Hospital of Shenzhen, Shenzhen, China; The University of Hong Kong, China

## Abstract

**Background:**

Genome-wide gene expression profile using deep sequencing technologies can drive the discovery of cancer biomarkers and therapeutic targets. Such efforts are often limited to profiling the expression signature of either mRNA or microRNA (miRNA) in a single type of cancer.

**Methodology:**

Here we provided an integrated analysis of the genome-wide mRNA and miRNA expression profiles of three different genitourinary cancers: carcinomas of the bladder, kidney and testis.

**Principal Findings:**

Our results highlight the general or cancer-specific roles of several genes and miRNAs that may serve as candidate oncogenes or suppressors of tumor development. Further comparative analyses at the systems level revealed that significant aberrations of the cell adhesion process, p53 signaling, calcium signaling, the ECM-receptor and cell cycle pathways, the DNA repair and replication processes and the immune and inflammatory response processes were the common hallmarks of human cancers. Gene sets showing testicular cancer-specific deregulation patterns were mainly implicated in processes related to male reproductive function, and general disruptions of multiple metabolic pathways and processes related to cell migration were the characteristic molecular events for renal and bladder cancer, respectively. Furthermore, we also demonstrated that tumors with the same histological origins and genes with similar functions tended to group together in a clustering analysis. By assessing the correlation between the expression of each miRNA and its targets, we determined that deregulation of ‘key’ miRNAs may result in the global aberration of one or more pathways or processes as a whole.

**Conclusions:**

This systematic analysis deciphered the molecular phenotypes of three genitourinary cancers and investigated their variations at the miRNA level simultaneously. Our results provided a valuable source for future studies and highlighted some promising genes, miRNAs, pathways and processes that may be useful for diagnostic or therapeutic applications.

## Introduction

Genitourinary neoplasms pose a large burden on human healthcare, with prostatic carcinoma ranked as the most predominant genitourinary tumors followed by bladder and kidney carcinoma in America [1], other types of genitourinary tumors are relatively uncommon. However, bladder, kidney, prostate and testicular carcinoma are the top four genitourinary tumors in China [2]. Bladder cancer is one of the most expensive cancers due to the necessity of life-long surveillance involving upper tract imaging, urinary cytology, and cystoscopy from diagnosis until the death of the patient [3]. Renal cell carcinoma (RCC) accounts for approximately 2% of all cancers, with a worldwide annual increase of 1.5–5.9% [4], [5]. Testicular cancer, on the other hand, represents between 1% and 1.5% of male neoplasms and 5% of urological tumors in general, and it is the most common cancer diagnosis in men between the age of 15 to 35 [6], [7]. Furthermore, epidemiological surveys indicate that there is a clear trend toward an increasing incidence of testicular cancer in most industrialized countries in North America, Europe and Oceania in recent decades, although substantial differences in the incidence rates are observed between neighboring countries [8].

Despite intensive efforts by many investigators over the past years, the exact mechanisms involved in the initiation and progression of these genitourinary cancers remain largely unclear. Transitional cell carcinoma (TCC) of the bladder is the most common (∼90%) histopathological type of bladder cancer. Many factors, including chromosomal anomalies, genetic polymorphisms, and genetic and epigenetic alterations, are thought to contribute to the tumorigenesis or progression of TCC [9]. Similarly, multiple environmental and genetic factors are also proposed to be associated with the development of clear cell renal cell carcinoma (ccRCC), which is the most common subtype (∼70%) of renal cell carcinoma [10]. In the case of testicular germ cell tumors (TGCT), the predominant (90–95%) subtype of testicular cancer, previous genome-wide expression analysis or targeted studies investigated the alpha-fetoprotein (AFP) mRNA level in atypical seminomas showed that seminomas and embryonal carcinomas may develop through common molecular mechanisms [11].

Our previous work on ccRCC demonstrated that simultaneously profiling the expression patterns of mRNAs and miRNAs in the same panel of cancer patients using a massively parallel sequencing platform (Illumina GA II) is a highly integrative and reproducible way of dissecting the molecular basis of human cancer [12]. Here we first generated the whole-genome expression profiles of protein coding genes in TCC and TGCT and characterized the expression patterns of miRNAs in TGCT. Combined with the results of our previous studies on ccRCC [12] and TCC [13], systematic comparison of the three different genitourinary cancers at both mRNA and miRNA levels in a total of 27 patient-matched tumor-normal tissue pairs identified multiple genes and miRNAs that were uniformly deregulated in all three genitourinary cancers or solely deregulated in a single kind of cancer. In addition, system-level or pathway-based analysis also highlighted the roles of common alterations in several cancer pathways and cancer-specific molecular events in tumor development.

## Results

### A snapshot of mRNA and miRNA profiling results generated in TGCT, TCC and ccRCC

Large numbers of transcripts showed consistent patterns of expression among the patients (seven TGCTs, ten TCCs and ten ccRCCs) with the same genitourinary cancer. Compared to the matched normal control, a total of 3181, 4321 and 4596 mRNAs were significantly differentially expressed (absolute value of log_2_ ratio ≥1, FDR≤0.01) in TGCT, TCC and ccRCC, respectively. The numbers of miRNAs significantly deregulated in those three cancers were 254, 226 and 118, respectively (absolute value of log_2_ ratio ≥1, *P*≤0.01). Statistical methods for determining the differentially expressed genes and miRNAs are described previously [12], and full lists of deregulated mRNAs and miRNAs can be found in Table S1 and S2, respectively. As reported in our previous studies [12], [13], the results of our whole-genome expression analysis correlated well with independent qPCR validation results, which is indicative of the good reliability of our sequencing approach and analytical pipeline.

**Figure 1:**
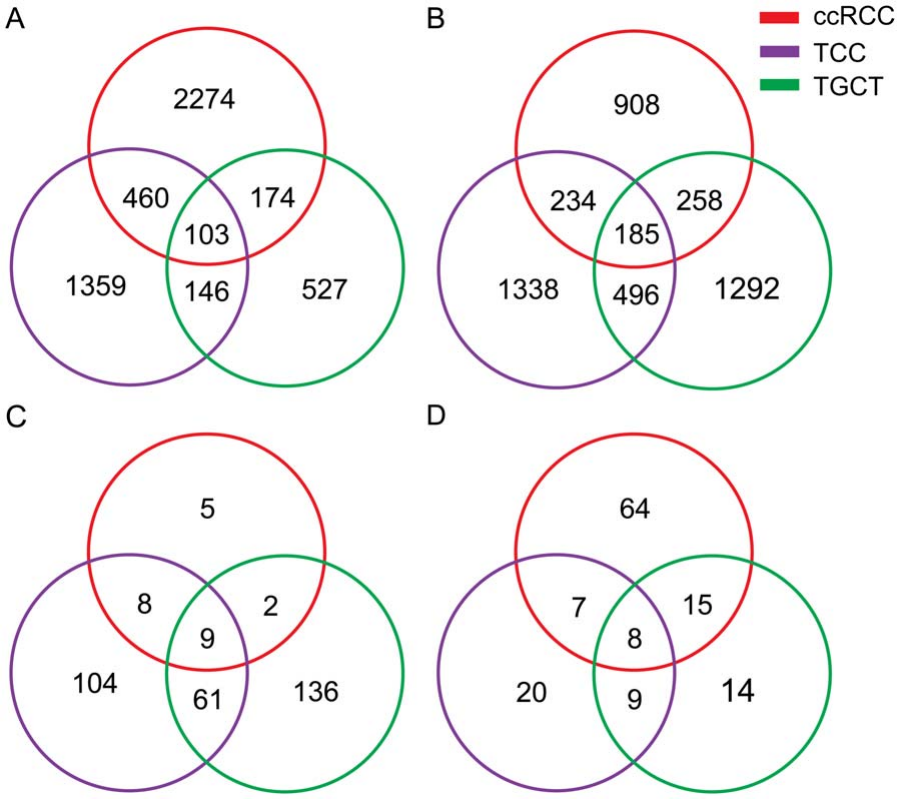
Deregulated genes and miRNAs in TCC, TGCT and ccRCC. Venn diagrams illustrate the overlapping relationship of the number of up-regulated genes (A), down-regulated genes (B), up-regulated miRNAs (C) and down-regulated miRNAs (D) among these cancers.

As shown in Figure 1, there were 103 and 185 genes that were detected to be up-regulated or down-regulated consistently in all of the three cancers, respectively, and only nine and eight miRNAs displayed consistent up- and down-regulation patterns, respectively. The numbers of genes and miRNAs that were deregulated (up or down) only in TGCT were 1,819 (527 up-regulated and 1,292 down-regulated) and 150, respectively. We also observed 2,697 genes and 124 miRNAs exhibiting significant expression changes only in TCC, and 3,182 genes and 69 miRNAs were deregulated solely in ccRCC. The remaining numbers in Figure 1 represent genes and miRNAs that were uniformly deregulated in two of the three genitourinary cancers.

### Pathway enrichment of genes significantly deregulated in the three cancers

**Figure 2:**
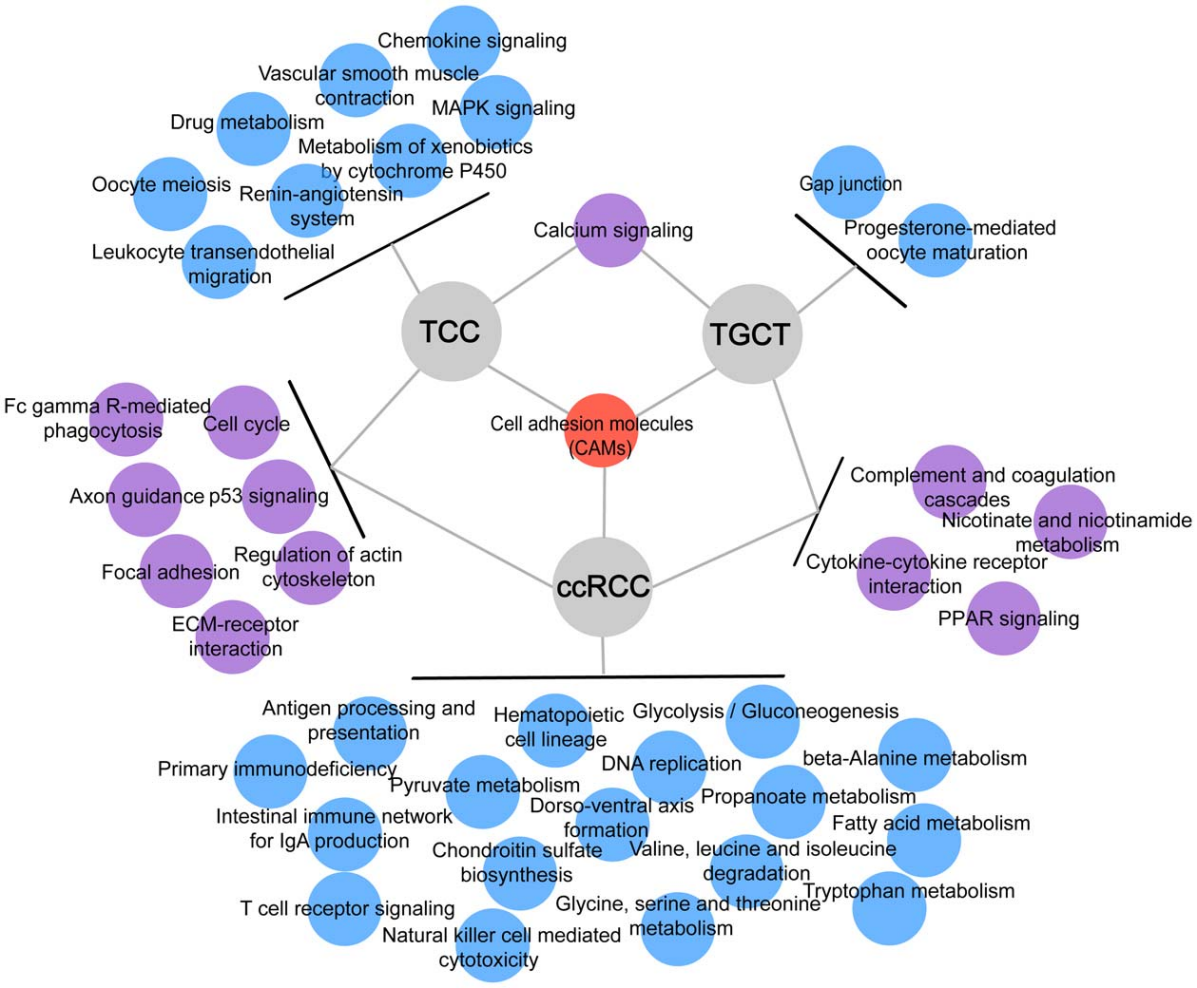
KEGG pathways significantly enriched with differentially expressed genes in TCC, TGCT and ccRCC. KEGG pathways significantly enriched (*P*≤0.05) with differentially expressed genes are illustrated, and the human disease pathways are manually removed. Pathways that were significantly enriched in all of three cancers are depicted in red, those enriched in two cancers are in yellow, and those enriched in only one cancer are in green.

All of the genes that were deregulated in each of the three cancers were used as inputs for the DAVID bioinformatics resource separately to identify the significantly deregulated pathways [14]. The resulting lists of KEGG pathways significantly enriched (*P*≤0.05) in deregulated genes in TGCT, TCC and ccRCC are listed in Table S3. As illustrated in Figure 2, the cell adhesion molecules (CAMs) pathway was affected in all the three cancers. Other pathways disrupted in two cancers included p53 signaling and the ECM-receptor and cell cycle pathways, which were disrupted in TCC and ccRCC, and the calcium signaling pathway, which was disrupted in TCC and TGCT. The cytokine-cytokine receptor interaction pathway was affected in both TGCT and ccRCC. Notably, the chemokine signaling pathway was also significantly enriched in deregulated genes in TCC, which indicated that perturbation of the normal function of cytokines might be a common molecular event in tumor development [15]. Other pathways that were affected only in a single type of cancer (for instance, MAPK signaling in TCC and glycolysis in ccRCC) are also illustrated in Figure 2. Interestingly, as noted in previous studies, global down-regulation of multiple metabolic pathways might be a characteristic phenotype of ccRCC, at least in comparison with the other two cancers profiled.

### Gene Ontology (GO) enrichment analysis of genes differentially expressed in the three cancers

Gene sets showing the same deregulated expression patterns in multiple cancers or genes showing cancer-specific expression deregulation are thought to be functionally important to the tumorigenic processes [16]. Thus, we examined all genes that exhibited the same deregulation patterns in two or three genitourinary cancers and all genes that were solely deregulated in only one cancer against the GO database to identify the significantly deregulated GO biological process terms (adjusted *P*≤0.05). The detailed results of the GO enrichment analysis are listed in Table S4. For the 288 genes that displayed the same deregulation patterns in all the three cancers, most of the extremely enriched GO biological terms were related to the cell cycle process (adjusted *P*≤5.0×10^-05^). Other important biological processes that were significantly enriched in genes deregulated in any two of the three cancers included the developmental process, cell adhesion and the cell differentiation process, which were affected in TGCT and TCC; the immune and inflammatory response process, which was affected in TGCT and ccRCC; and the DNA replication and repair processes, which was affected in TCC and ccRCC. Remarkably, we also observed that gene sets showing TGCT-specific deregulation patterns were mainly implicated in processes related to male reproductive function, such as spermatogenesis, fertilization and spermatid development [17]. Genes solely deregulated in TCC were enriched in biological processes associated with tumor invasiveness and metastasis, such as the developmental process, cell (-matrix) adhesion, cell differentiation, cell motion and cell junction organization [18]. Many genes that were deregulated only in ccRCC were implicated in processes associated with the immune response and transmembrane transport of relatively small molecules.

### Gene Expression Correlates with Tumor Types

**Figure 3:**
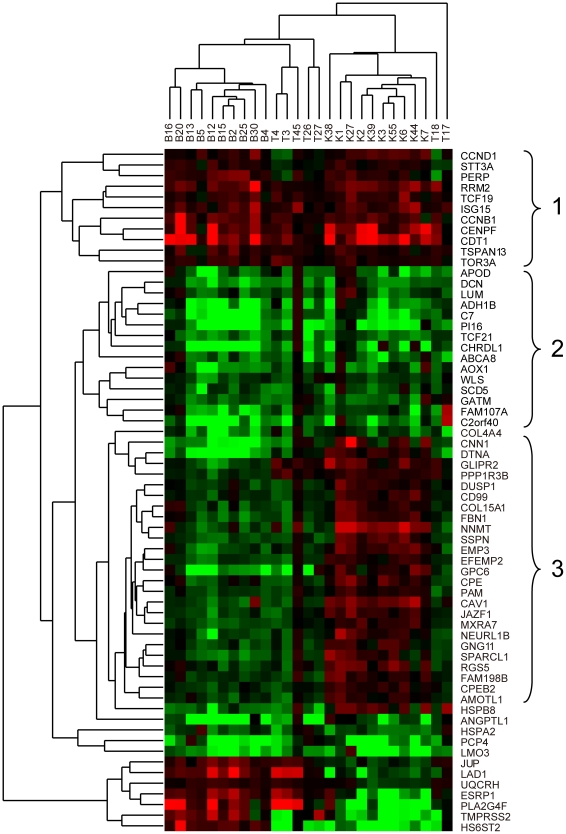
Unsupervised hierarchical clustering of expression data from 64 genes. The differential expression value matrix of 64 genes with absolute value of log_2_ ratio ≥1 and FDR≤0.01 in at least 75% of 27 patients was used to perform unsupervised hierarchical clustering. The different tumor types (labeled in top; B, TCC; T, TGCT; K, ccRCC) were clustered by the up-regulation (red) and down-regulation (green) patterns of corresponding genes (listed vertically by their official gene symbol).

We next studied the differences in gene expression profiles that could be used to distinguish between, or give biological insights into the molecular disparities of the tumors with distinct histological origins. Of the 596 genes that were deregulated in all the three cancers (Table S5), 64 were differentially expressed (absolute value of log_2_ ratio ≥1, FDR ≤0.01) in at least 75% of the 27 patients. As shown in Figure 3, unsupervised hierarchical clustering of the tumor samples based on the relative expression levels of these 64 genes revealed that tumors with the same histological origin generally tended to group together (except TGCT T17 and T18) and that these genes could be divided into five major clusters showing different expression patterns among the tumors. Genes in cluster 1 and cluster 2 were predominantly up-regulated and down-regulated in the majority of tumors sequenced, respectively. In contrast, genes grouped in cluster 3 were up-regulated in ccRCC but down-expressed in TCC and TGCT.

### Deregulated expression patterns of miRNAs in the three genitourinary cancers

In this comparative study, we observed that over half (17/31) of the miRNAs that were aberrantly expressed in all the three cancers exhibited consistent expression patterns (Figure 1C and 1D). Many of these miRNAs (such as miR-142-3p, miR-155, miR-21 and miR-210) that were known to function as ‘oncogenes’ in previous studies were observed to be uniformly up-regulated, while other potential ‘tumor suppressors’ such as let-7c and miR-214 were down-regulated in our study (reviewed by Ramiro Garzon et al. [19] and Aurora Esquela-Kerscher et al. [20]). A large proportion of the deregulated miRNAs also showed cancer-specific expression patterns (Figure 1C and 1D). Consistent with other studies on TGCT [21], [22], miR-372 and miR-373 have been suggested to act as oncogenes, and were found to be up-regulated solely in TGCT as well as the star form miR-373* in our study, further confirming their special roles in TGCT development. miRNAs solely deregulated in the other two cancers included miR-143 and miR-195, which were down-regulated in TCC. miR-122, a previously recognized tumor suppressor that had been shown to be specific to liver cancer [23], was up-regulated in ccRCC.

As shown in Table 1, we also noticed that a significant number of miRNA families were deregulated in two or three types of cancers. Multiple members of the miR-8 family, whose associations with human cancers had been reported repeatedly [24], [25], exhibited varied expression patterns in these three cancers. Other miRNA families (such as the miR-199, miR-17 and miR-506 families) that have not received previous annotation in cancer studies were also observed to be deregulated either consistently or inconsistently in two or three tumor types in this study (Table 1). Interestingly, we identified three peculiar miRNA families or clusters that exhibited TGCT-specific up-regulated expression patterns (Table S2). 42 members of the miR-515 family (miR-515–527) and eight members of the miR-302–367 cluster (miR-302a, miR-302a*, miR-302b, miR-302b* miR-302c, miR-302d, miR-302d* and miR-367) were only up-regulated in TGCT and not in the other two cancers, and three members of the miR-105-767 cluster (miR-105, miR-105* and miR-767-5p) were ranked as the top ten up-regulated miRNAs in TGCT.

**Table 1:** miRNA families deregulated in TCC, TGCT, ccRCC.

Family	Deregulated members	TCC^a^	TGCT	ccRCC
mir-8	miR-141	3.28	1.98	−2.06
	miR-200a	3.05	−1.22	−1.14
	miR-200b	2.98	−2.39	−1.59
	miR-200b*	3.20	−1.55	−1.33
	miR-200c	2.97	2.47	−3.25
	miR-429	3.30	-	−1.88
mir-199	miR-199a-3p	−1.72	−1.20	−1.24
	miR-199a-5p	−1.80	−1.26	−1.30
	miR-199b-3p	−1.72	−1.20	−1.24
mir-17	miR-106b	2.10	1.47	-
	miR-106b*	1.09	1.27	-
	miR-18a	2.56	1.87	-
	miR-18a*	1.34	1.18	-
	miR-20a	1.82	1.22	-
	miR-20a*	1.12	1.41	-
	miR-93	1.94	1.48	-
mir-506	miR-506	-	−1.98	−3.30
	miR-508-3p	-	−1.21	−3.83
	miR-509-5p	-	−1.26	−3.65
	miR-510	-	−1.71	−1.51
	miR-513c	-	−1.14	−1.40
	miR-514	-	−1.92	−3.63

a Log_2_ ratio value (tumor versus normal tissue), *P*≤0.01.

### Pearson's correlation between the expression levels of differentially expressed miRNAs and their potential target genes

We then investigated the biological relevance of the significantly deregulated miRNAs by evaluating the correlation between the expression levels of each miRNA and all of its predicted target genes. Significant correlations (*P*≤0.05) were found between 77, 196 and 444 miRNA-mRNA pairs in TGCT, TCC and ccRCC, respectively (Table S6). The GO biological processes that were enriched with these significantly correlated target genes were determined using DAVID, and the enriched GO categories are listed in Table S7 for TGCT, TCC and ccRCC, respectively. Multiple core regulatory processes or signaling pathways that are likely to be of functional relevance to tumor development, including the developmental process, the JAK-STAT cascade, cell differentiation, cell motion and other processes associated with cell death or apoptosis regulation, were all predicted to be under the potential influence of deregulated miRNAs.

By careful examination of the individual miRNAs that negatively correlated with the expression levels of their target genes, we found that miR-367 (belonging to the miR-302-367 cluster mentioned above) was up-regulated solely in TGCT and its predicted target *CDKN1C*, an imprinted gene which is characterized by frequent loss of imprinting in esophageal and breast cancers, was also down-regulated in TGCT (*r* = -0.59, *P* = 0.024) [26], [27]. Another miRNA, miR-195, was down-regulated and its target *MAP7*, which is predominantly expressed in cells of epithelial origin and is able to stabilize microtubules, was observed to be up-regulated in TCC (*r* = −0.48, *P* = 0.031) [28].

As expected, the expression levels of some miRNAs were significantly correlated with multiple target genes. 43 predicted target genes under the regulation of the up-regulated miR-19a/b were all down-regulated in TCC, and 11 of them were predicted to be regulated by both miR-19a and miR-19b (Table S6). In addition, three miRNAs miR-20b, miR-363 and miR-30c that were all solely down-regulated in ccRCC also showed significant correlation with the expression levels of 23, 25 and 37 of their predicted target genes, respectively (Table S6). Notably, the correlated target genes of miR-20b were mainly implicated in the regulation of metabolic processes (adjusted *P* = 0.037) and three of the targets (*JAK1*, *CCND2* and *SPRED1*) participate in regulating the JAK-STAT signaling pathway. However, no enriched biological process was found in the correlated target gene sets of miR-30c and miR-363.

## Discussion

The present study provided an integrated overview of three genitourinary cancers based on their whole-genome expression profiles of both mRNA and miRNA. All matched tumor-normal sample pairs from the 27 cancer patients were prepared and sequenced using the same protocols on the same platform, and the resulting raw sequencing data were also submitted to similar preliminary analyses [12]. Thus, we assumed that technical variations due to inter-laboratory or inter-platform differences should be minimal or negligible in this study. Our results revealed that the comparative analysis of different cancers could provide additional in-depth insights into the system-level molecular mechanisms of tumorigenesis that are otherwise relatively difficult to be deciphered by focusing on only a single type of cancer. Furthermore, we obtained the expression profiles of miRNAs and protein-coding genes in all 27 patients. Integrative analysis of mRNA and miRNA expression profiles facilitated the identification of deregulated biological processes or pathways that may be under the regulation of miRNAs, which in turn could serve as ideal therapeutic targets [29].

Research over recent decades has suggested that transformation of normal cells into malignant cancers may be governed by several common rules [30]. Our comparative analysis of the three different genitourinary cancers also revealed the common physiological changes occurring in tumor cells. Significant deregulation of developmental and differentiation processes and the pathways controlling cell cycle in two or more cancers indicated that the dedifferentiated tumor cells were insensitive to the inhibitory (anti-growth) signals that would normally block the entry into an active cell cycle state. Deregulation of the p53 signaling pathways may facilitate both angiogenesis and resistance to apoptosis [31]. Aberration of the CAMs pathway and ECM receptors enables cancer cells to escape their primary tumor masses, invade adjacent tissues and colonize elsewhere [32], [33]. Additionally, as demonstrated in our study, frequent deregulation of the cytokine-related pathways as well as the immune and inflammatory response processes is another common hallmark of human cancer [34]. For many solid tumors, cytokines, together with CAMs, play important roles in the induction of antitumor immune responses and tumor rejection in the tumor microenvironment where immune and malignant cells interact [35]. Moreover, recent emerging data suggested that cancer-related inflammation contributes to the proliferation and survival of tumor cells and linked this inflammation to the therapeutic response and prognosis of cancer patients [36].

Systematic comparisons of three genitourinary cancers also demonstrated that a large number of deregulated genes were enriched in specific pathways or processes that characterize the distinct biological behaviors of each cancer. Gene sets solely deregulated in TGCT are mainly implicated in male reproduction-related processes such as spermatogenesis, spermatid development and fertilization, indicating that normal testicular function had been lost during the conversion of normal somatic cells into tumor cells. Meanwhile, global down-regulation of multiple metabolic pathways and significant deregulation of biological processes related to blood coagulation, lipid and ion transport were only observed in ccRCC. As reported previously, disorder of intravascular coagulation may result in renal lesions [37]. The unique expression profile of TCC was characterized by significant deregulation of processes linked to tumor invasiveness and metastasis. In accordance with its molecular phenotypes, TCC also behaves uniquely in terms of its clinical features and prognosis. It is estimated that approximately 70% of TCC patients present superficial non-muscle-invasive tumors that are not life-threatening but tend to recur; the remaining cases presenting muscle-invasive tumors are usually at a high risk of death associated with distant metastasis [38].

Classification of cancer based on the expression patterns of key molecular markers that can be used for cancer diagnosis, prognosis and prediction had been widely described and applied in previous studies [39]. In this study, hierarchical clustering of genes deregulated in the majority (over 75%) of patients profiled can generally classify the tumors into groups with the same histopathological manifestations (Figure 3). Genes in cluster 1 are dominated by oncogenes that are up-regulated in most tumors, such as *CCND1*, *PERP* and *RRM2* [40], [41], [42]. Moreover, *TCF19* encodes a transcription factor that is assumed to play a pivotal role in regulating the expression of target genes. The gene product of *CCNB1* (cyclin B1) and cdc2 form the maturation-promoting complex, which is important for stimulating mitosis [43]. The product of *CENPF* may play a role in chromosome segregation during mitosis [44] and can be used as a valuable marker of nasopharyngeal carcinoma progression [45]. In contrast, genes belonging to cluster 2 significantly lost their expression in most of the genitourinary neoplasms profiled. Many genes grouped in cluster 2 are recognized as candidate tumor suppressors in previous studies. Methylation-associated silencing of *FAM107A* was identified in the three genitourinary tumors [46]. The protein product of *DCN* (decorin) is capable of suppressing the growth of various tumor cells [47], [48], [49]. *TCF21* was aberrantly methylated and silenced in head and neck squamous cell carcinoma and non-small-cell lung cancer [50]; *C2orf40* was hypermethylated and transcriptional silenced in colorectal carcinoma and glioma, and its over-expression led to a significant decrease in cell growth [51]. Overall, though most of genes mentioned above were well-known for other cancers, their abnormalities in genitourinary tumors were seldom reported before. Our findings highlighted their general effects in TCC, TGCT and ccRCC. Furthermore, as genes with similar expression patterns or functions tending to group together, other genes (e.g., *TCF19*) in cluster 1 and 2 may also be responsible for important functions in these three cancers and thus are worthy of further attention in future studies.

In addition, we also identified a subset of genes (cluster 3) that were up-regulated in ccRCC but down-regulated in TCC and TGCT. The expression of *CNN1* is a late stage differentiation marker of smooth muscle cells, and a decrease in *CNN1* is associated with underdeveloped renal tumor vessels that lack integrity [52], [53]. Three genes (*AMOTL1*, *CAV1* and *COL15A1*) in this class were functionally associated with the angiogenesis process. *AMOTL1* controls cell migration and cell-cell adhesion in vivo during angiogenesis [54]. *CAV1* enhances the formation of endothelial capillaries [55] and the silencing of its expression by antisense oligodeoxynucleotides impairs the angiogenesis process *in vitro* and *in vivo* [56]. Moreover, over-expression of *CAV1* in ccRCC had been documented previously and is thought to be important for the progression of ccRCC [57], [58]. Vascular systems in different tumors are exceptionally variable in their sizes, shapes and branching patterns and are usually not organized in the conventional hierarchy of arterioles, capillaries and venules [59]. Renal cell carcinoma is a vascular-rich tumor, and we envisioned the possibility that the larger the tumor mass is, the more complex vascular system the tumor needs for nutrition. Thus, adaptive over-expression of angiogenesis-related genes should be essential for the proliferation of tumor cells within the huge mass of ccRCC.

miRNAs are an important class of small non-coding RNAs that are capable of regulating the temporal and tissue-specific expression patterns of protein coding genes at the post-transcriptional level, blocking translation and/or leading to mRNA degradation [60]. miRNAs play active roles in tumorigenesis by regulating the expression of their target genes, resulting in an aberration of multiple core biological processes or pathways [20], [61]. Our analysis identified a number of annotated cancer-associated miRNAs such as miR-142-3p, miR-155, miR-21, miR-210, let-7c and miR-214 exhibiting consistent deregulation in TCC, TGCT and ccRCC, which may suggest their general effects on human cancer development. Some miRNA families or clusters as a whole were also significantly deregulated in our study (Table 1). The observed co-expression patterns for these miRNA families or clusters were expected, as other studies had proposed that most of the known miRNAs are tandemly clustered and transcribed as polycistronic primary transcripts [62], [63]. miR-8 (also known as “miR-200”) family exhibited different patterns in TCC, TGCT and ccRCC in our study. Down-regulation of miR-8 family were identified in multiple cancers like breast, ovarian and pancreatic neuroendocrine tumors, and inhibition of miR-8 family induces the epithelial-to-mesenchymal transition (EMT), which is viewed as an essential early step in tumor metastasis [24], [25], [64]. Interestingly, over-expression of miR-8 family has been also shown to enhance mouse breast cancer cell colonization to form distant metastases [65]. In short, miR-8 family may play different roles in different human cancers, and our data also suggested their diverse roles in the genitourinary cancers.

Integrative correlation analysis of the expression levels of miRNAs and their predicted target genes in the three cancers further highlighted the effects of certain miRNAs on tumor development. Of the large number of targets whose expression levels correlated negatively with miR-19a and/or miR-19b in TCC (Table S6), many genes were well known to be associated with cancer based on previous functional studies. For instance, the product of target gene *ARRDC3* can bind and degrade the ITGβ4 protein, which affects the proliferation, migration and invasion of breast cancer cells *in vitro* [66]. The expression of another target, *CBX7*, decreases with malignancy grade and neoplasia stage in thyroid cancer [67] and is associated with a more aggressive phenotype in pancreatic cancer [68]. Thus, we believed that down-regulation of miR-19a and miR-19b may also promote the tumorigenic process of TCC. Another miRNA worthy of notice is miR-20b in ccRCC, which targeted 3 genes (*JAK1*, *CCND2* and *SPRED1*) participating in JAK-STAT signaling. Down-regulation of miR-20b may therefore lead to the activation of the JAK-STAT pathway, which has been documented in many cancer studies [69], [70], [71], [72]. Strikingly, the down-regulated targets of miR-20b have been mainly implicated in many metabolic processes (adjusted *P* = 0.037), which may, in part, account for the characteristic phenotype of ccRCC (global deregulation of multiple metabolic pathways in ccRCC as shown in Figure 2). In short, the aberrant expression of miR-19a/b and miR-20b may result in the alterations of many downstream activities, and they may therefore they may serve as the ideal candidates for future therapeutics development.

In summary, we present a comparative analysis of the whole-genome expression profiles of both mRNA and miRNA in three different genitourinary cancers, TCC, TGCT and ccRCC. Our findings reveal the common changes in a few conserved biological processes or pathways in human cancer and highlight the effects of several commonly deregulated genes and miRNAs on tumor development. In addition, our analysis also identified a number of pathways, processes and individual markers that showed cancer-specific expression changes. Taken together, this integrated comparative study generated a system-level sketch of the molecular phenotypes of TCC, TGCT and ccRCC and simultaneously investigated the variations of miRNA levels in the each matched cancer patient. Our study can serve as a valuable source for future studies on a given cancer or different carcinomas, and some of the highlighted genes, miRNAs, pathways or processes may be useful for diagnostic or therapeutic purposes.

## Materials and Methods

### Clinical sample collection

Written informed consent from all patients was obtained, and this series of studies was reviewed and approved by Institutional Ethics Committees of Peking University Shenzhen Hospital (Shenzhen, China). All tumor and matched normal adjacent tissues in this study were obtained from the biobank (CXC201005260001A) of the Urinogenital Cancer Genomics Consortium (UCGC) in China. Information on the 27 patients (10 TCC, 7 TGCT and 10 ccRCC) analyzed is summarized in Table S8. Specimens were deposited in RNALater (Qiagen, Germany) or snap-frozen in liquid nitrogen and subsequently stored at −80°C. Hematoxylin-eosin (HE)-stained sections were examined for tumor cell types and percentages of cells; tumor tissues containing more than 80% tumor cells were selected for further study. The matched normal adjacent tissues were defined as tissues located at least 1.0, 1.0 and 2.0 cm apart from the visible tumor lesions in TGCT, TCC and ccRCC, respectively. Representative histological examination results of the tumors and matched normal adjacent tissues of TCC, TGCT and ccRCC are showed in Figure S1.

### mRNA and miRNA sequencing and preliminary analyses of raw data

Total RNA was extracted from normal adjacent tissues and tumor tissues of each patients studied. Four and five micrograms of total RNA were subjected to mRNA and miRNA sequencing library preparation, respectively, before they were sequenced using the Illumina GAII platform according to manufacturer's instructions (Illumina Inc, USA). Manipulation of raw sequencing data, reference alignment and determination of differentially expressed mRNA and miRNA were all performed as described previously [12]. Briefly, the expression fold change (tumor versus normal) for each gene and miRNA was calculated as the log_2_ ratio using the normalized TPM (transcripts per million reads) values. Subsequently, we performed a rigorous significance test to determine the differentially expressed genes and miRNAs [73]. Additionally considering the large amount of mRNA transcripts, the resulting *P*-values (*P*) were corrected for multiple tests using the FDR (false discovery rate) adjustments [74].

### Hierarchical clustering analysis of the mRNA profile

Of the 596 genes that were deregulated in all three cancers, only 64 genes were differentially expressed (absolute value of log_2_ ratio ≥1, FDR ≤0.01) in at least 75% of 27 patients. The uncentered correlation was calculated to cluster genes and tumor samples based on the relative expression of these 64 genes using the average linkage hierarchical clustering algorithm applied in the cluster program [75].

### Target prediction of differentially expressed miRNAs

We investigated the biological relevance of differentially expressed miRNAs through their regulation on target genes. First, a putative target gene set was generated according to the intersect result of any two predicted algorithms of DIANA-microT 3.0 [76], TargetScan 5.1 [77], and PicTar [78]. Second, Pearson correlation analysis was performed using R (http://www.R-project.org) to determine whether the expression levels of each miRNA and its mRNA targets were negatively correlated (*P*≤0.05). Finally, the putative targets confirmed by the correlation analysis were subjected to further analysis.

### KEGG pathway and Gene Ontology analysis of differentially expressed genes and miRNA targets

Different sets of differentially expressed genes and miRNA targets were used as input for the DAVID bioinformatics resource. KEGG pathways with EASE values ≤0.05 (*P*-values from modified Fisher's exact test; more information can be found in http://david.abcc.ncifcrf.gov/) were deemed to be statistically significantly deviated from the expected distribution, and thus, the corresponding pathways were enriched with the deregulated genes in question. Considering the large amount and complex branch structure of GO biological processes, we used a significance threshold *P*-value adjusted by Benjamini of 0.05 for biological process terms to control the false discovery rate [74].

## Supporting Information

Figure S1 **HE staining of tumor and normal adjacent tissues from TCC, TGCT and ccRCC.**
(PDF)

Table S1 **Differentially expressed genes in TGCT, TCC and ccRCC.**
(XLS)

Table S2 **Differentially expressed miRNAs in TGCT, TCC and ccRCC.**
(XLS)

Table S3 **KEGG pathways enriched in differentially expressed genes in TGCT, TCC and ccRCC.**
(XLS)

Table S4 **Biological process analysis of gene sets that were deregulated uniformly in all cancers or only in TCC, TGCT or ccRCC.**
(XLS)

Table S5 **Genes deregulated commonly in TCC, TGCT and ccRCC.**
(XLS)

Table S6 **Target prediction of differentially expressed miRNA in TGCT, TCC and ccRCC.**
(XLS)

Table S7 **Biological processes involvement of target genes of differentially expressed miRNAs.**
(XLS)

Table S8 **Clinical information on the 27 patients in our study.**
(XLS)
