# Supplementary material for: Comparative mRNA and microRNA Expression Profiling of Three Genitourinary Cancers Reveals Common Hallmarks and Cancer-Specific Molecular Events
Source: PLoS One. 2011 Jul 25;6(7):e22570. doi: 10.1371/journal.pone.0022570 (PMC3143156; doi:10.1371/journal.pone.0022570)
Supplement: Figure S1 — HE staining of tumor and normal adjacent tissues from TCC, TGCT and ccRCC. (PDF) [file pone.0022570.s001.pdf]

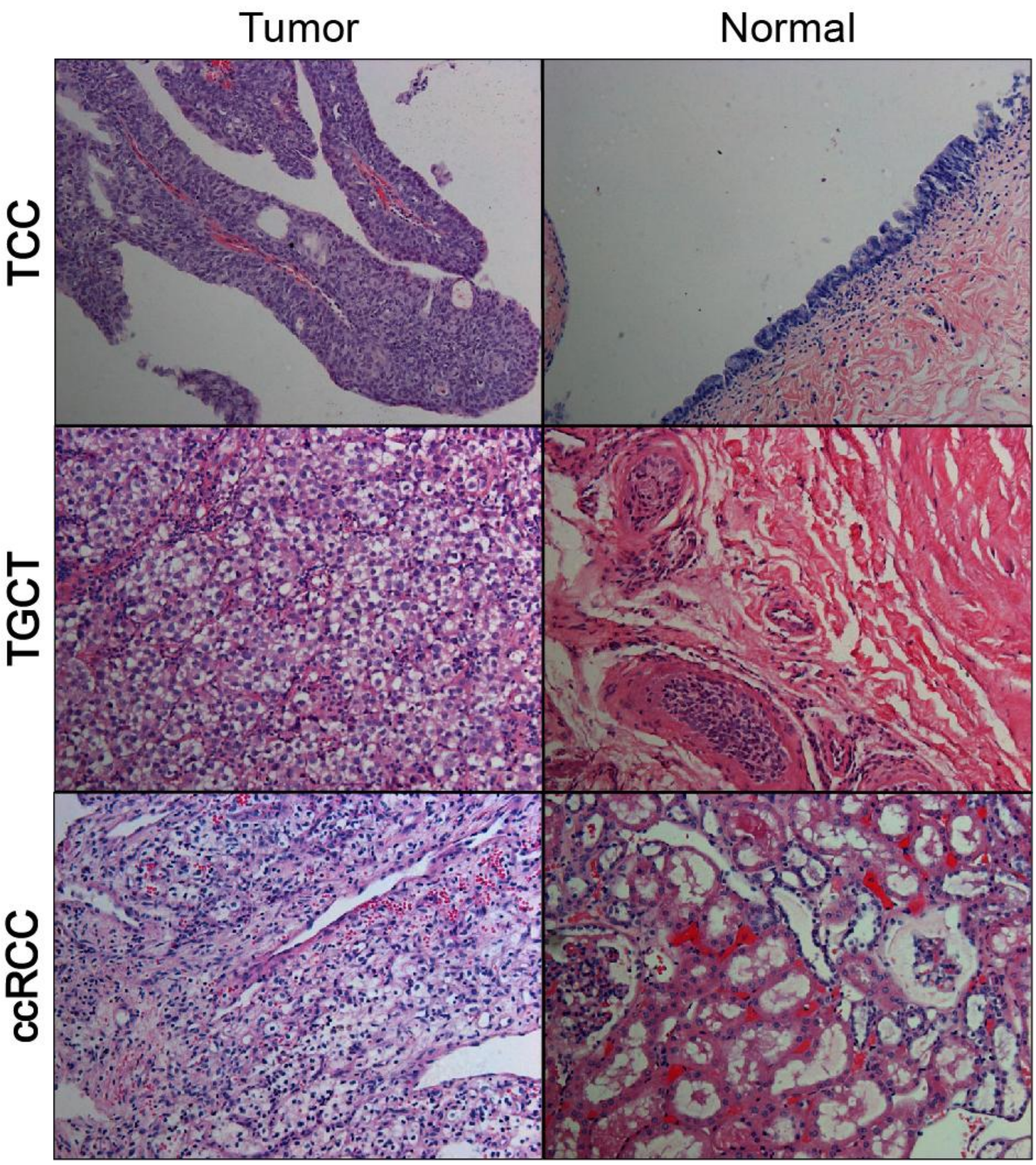

Figure S1. HE staining of tumor and normal adjacent tissues from TCC, TGCT and ccRCC. Figure S1 illustrated the representative tumor (left) and normal (right) adjacent tissue sections form TCC, TGCT and ccRCC, respectively.
